# Crystal structure of mandipropamid

**DOI:** 10.1107/S2056989015016643

**Published:** 2015-09-12

**Authors:** Hyunjin Park, Jineun Kim, Gihaeng Kang, Tae Ho Kim

**Affiliations:** a Department of Chemistry and Research Institute of Natural Sciences, Gyeongsang National University, Jinju 52828, Republic of Korea

**Keywords:** crystal structure, conformation, hydrogen bonding, C—H⋯π inter­actions, amide fungicide

## Abstract

In the title compound, C_23_H_22_ClNO_4_ (systematic name: (*RS*)-2-(4-chloro­phen­yl)-*N*-{2-[3-meth­oxy-4-(prop-2-yn-1-yl­oxy)phen­yl]eth­yl}-2-(prop-2-yn­yloxy)acetamide), an amide fungicide, the dihedral angle between the chloro­benzene and benzene rings is 65.36 (6)°. In the crystal, N—H⋯O hydrogen bonds lead to zigzag supra­molecular chains along the *c* axis (glide symmetry). These are connected into layers by C—H⋯O and C—H⋯π inter­actions; the layers stack along the *a* axis with no specific inter­molecular inter­actions between them.

## Related literature   

For information on the fungicidal properties of the title compound, see: Zhang *et al.* (2014 ▸). For a related crystal structure, see: Davis & Healy (2008 ▸).
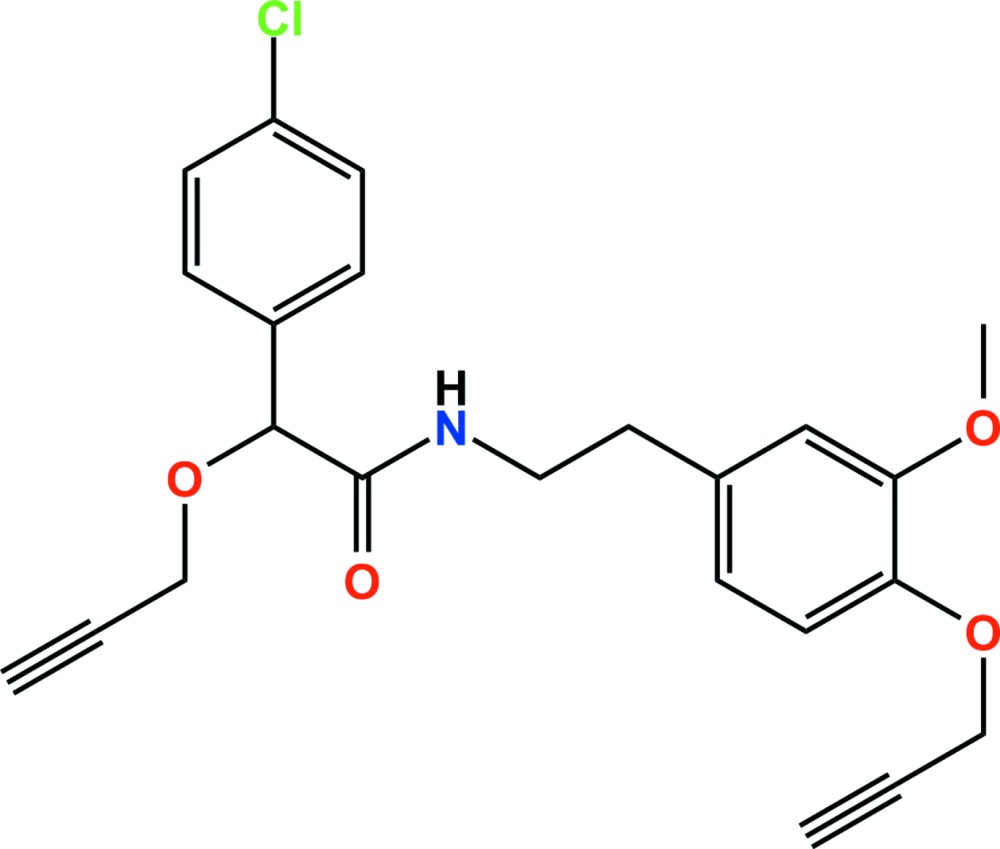


## Experimental   

### Crystal data  

C_23_H_22_ClNO_4_
*M* *_r_* = 411.86
Monoclinic, 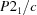

*a* = 26.3733 (17) Å
*b* = 9.4740 (6) Å
*c* = 8.4882 (5) Å
β = 91.013 (3)°
*V* = 2120.5 (2) Å^3^
*Z* = 4
Mo *K*α radiation
μ = 0.21 mm^−1^
*T* = 173 K
0.22 × 0.15 × 0.10 mm

### Data collection  

Bruker APEXII CCD diffractometer
Absorption correction: multi-scan (*SADABS*; Bruker, 2013 ▸) *T*_min_ = 0.722, *T*_max_ = 0.746
36727 measured reflections
4885 independent reflections
3686 reflections with *I* > 2σ(*I*)
*R*_int_ = 0.039

### Refinement  

*R*[*F* ^2^ > 2σ(*F* ^2^)] = 0.052
*wR*(*F* ^2^) = 0.131
*S* = 1.04
4885 reflections
263 parameters
H-atom parameters constrained
Δρ_max_ = 0.82 e Å^−3^
Δρ_min_ = −0.29 e Å^−3^

### 

Data collection: *APEX2* (Bruker, 2013 ▸); cell refinement: *SAINT* (Bruker, 2013 ▸); data reduction: *SAINT*; program(s) used to solve structure: *SHELXS97* (Sheldrick, 2008 ▸); program(s) used to refine structure: *SHELXL2013* (Sheldrick, 2015 ▸); molecular graphics: *DIAMOND* (Brandenburg, 2010 ▸); software used to prepare material for publication: *SHELXTL* (Sheldrick, 2008 ▸).

## Supplementary Material

Crystal structure: contains datablock(s) global, I. DOI: 10.1107/S2056989015016643/tk5382sup1.cif

Structure factors: contains datablock(s) I. DOI: 10.1107/S2056989015016643/tk5382Isup2.hkl

Supporting information file. DOI: 10.1107/S2056989015016643/tk5382Isup3.cml

**Fig. 1.**
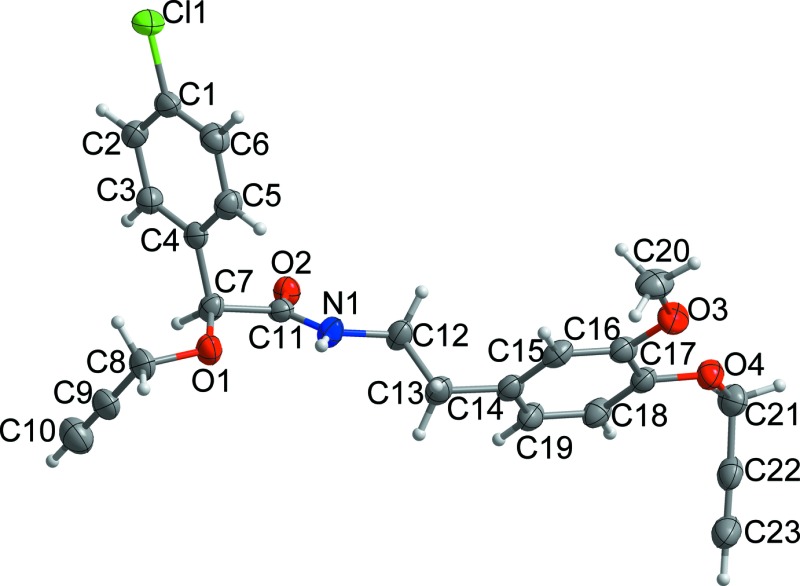
The molecular structure of the title compound with the atom-numbering scheme. Displacement ellipsoids are drawn at the 50% probability level. H atoms are shown as small spheres of arbitrary radius.

**Fig. 2.**
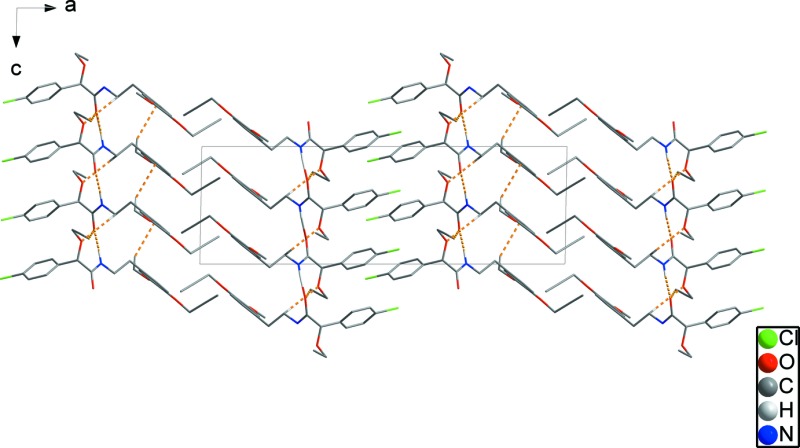
Crystal packing viewed along the b axis. The hydrogen bonds are shown as dashed lines.

. DOI: 10.1107/S2056989015016643/tk5382fig1.tif
The mol­ecular structure of the title compound with the atom-numbering scheme. Displacement ellipsoids are drawn at the 50% probability level. H atoms are shown as small spheres of arbitrary radius.

b . DOI: 10.1107/S2056989015016643/tk5382fig2.tif
Crystal packing viewed along the *b* axis. The hydrogen bonds are shown as dashed lines.

CCDC reference: 1422569

Additional supporting information:  crystallographic information; 3D view; checkCIF report

## Figures and Tables

**Table 1:** Hydrogen-bond geometry (, ) *Cg*2 is the centroid of the C14C19 ring.

*D*H*A*	*D*H	H*A*	*D* *A*	*D*H*A*
N1H1*N*O2^i^	0.88	2.04	2.850(2)	152
C10H10O2^ii^	0.95	2.35	3.218(3)	152
C12H12*A*O1^iii^	0.99	2.53	3.232(3)	128
C20H20*C* *Cg*2^iv^	0.98	2.84	3.709(3)	148

Symmetry codes: (i) 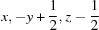; (ii) 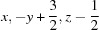; (iii) 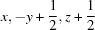; (iv) 
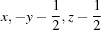
.

## supplementary crystallographic information

### S1. Comment

Mandipropamid [systematic name: (*RS*)-2-(4-chlorophenyl)-*N*-[3-methoxy-4-(prop-2-ynyloxy)phenethyl]-2-(prop-2-ynyloxy)acetamide] is a the amide fungicide that is used on potato, tomato, pepper, grape, watermelon, and litchi. (Zhang *et al.*, 2014). The dihedral angle between the chlorobenzene and the benzene rings is 65.36 (6)°. All bond lengths and bond angles are normal and comparable to those observed in a similar crystal structure (Davis & Healy, 2008).

In the crystal structure (Fig. 2), N–H···O and C–H···O hydrogen bonds and weak C–H···π interactions (Table 1) link adjacent molecules.

### S2. Experimental

The title compound was purchased from Dr. Ehrenstorfer GmbH. Slow evaporation of its solution in CH_3_CN gave single crystals suitable for X-ray analysis.

### S3. Refinement

All H-atoms were positioned geometrically and refined using a riding model with d(N—H) = 0.88 Å, *U*_iso_ = 1.2*U*_eq_(C) for N—H, and d(C—H) = 0.95–1.00 Å, *U*_iso_ = 1.2–1.5*U*_eq_(C) for C—H. Owing to poor agreement, one reflection, i.e. ( 1 0 0), was omitted from the final cycles of refinement.

### Crystal data

**Table:**

C_23_H_22_ClNO_4_	*F*(000) = 864
*M**_r_* = 411.86	*D*_x_ = 1.290 Mg m^−^^3^
Monoclinic, *P*2_1_/*c*	Mo *K*α radiation, λ = 0.71073 Å
*a* = 26.3733 (17) Å	Cell parameters from 9957 reflections
*b* = 9.4740 (6) Å	θ = 2.3–26.3°
*c* = 8.4882 (5) Å	µ = 0.21 mm^−^^1^
β = 91.013 (3)°	*T* = 173 K
*V* = 2120.5 (2) Å^3^	Plate, colourless
*Z* = 4	0.22 × 0.15 × 0.10 mm

### Data collection

**Table:**

Bruker APEXII CCD diffractometer	3686 reflections with *I* > 2σ(*I*)
φ and ω scans	*R*_int_ = 0.039
Absorption correction: multi-scan (*SADABS*; Bruker, 2013)	θ_max_ = 27.6°, θ_min_ = 2.3°
*T*_min_ = 0.722, *T*_max_ = 0.746	*h* = −33→34
36727 measured reflections	*k* = −12→12
4885 independent reflections	*l* = −11→10

### Refinement

**Table:**

Refinement on *F*^2^	0 restraints
Least-squares matrix: full	Hydrogen site location: inferred from neighbouring sites
*R*[*F*^2^ > 2σ(*F*^2^)] = 0.052	H-atom parameters constrained
*wR*(*F*^2^) = 0.131	*w* = 1/[σ^2^(*F*_o_^2^) + (0.0444*P*)^2^ + 1.677*P*] where *P* = (*F*_o_^2^ + 2*F*_c_^2^)/3
*S* = 1.04	(Δ/σ)_max_ = 0.001
4885 reflections	Δρ_max_ = 0.82 e Å^−^^3^
263 parameters	Δρ_min_ = −0.29 e Å^−^^3^

### Special details

**Table:**

Geometry. All e.s.d.'s (except the e.s.d. in the dihedral angle between two l.s. planes) are estimated using the full covariance matrix. The cell e.s.d.'s are taken into account individually in the estimation of e.s.d.'s in distances, angles and torsion angles; correlations between e.s.d.'s in cell parameters are only used when they are defined by crystal symmetry. An approximate (isotropic) treatment of cell e.s.d.'s is used for estimating e.s.d.'s involving l.s. planes.

### Fractional atomic coordinates and isotropic or equivalent isotropic displacement parameters (Å^2^)

**Table:**

	*x*	*y*	*z*	*U*_iso_*/*U*_eq_	
Cl1	0.45595 (2)	0.22978 (7)	0.12537 (9)	0.0543 (2)	
O1	0.67498 (6)	0.42193 (16)	−0.19373 (17)	0.0376 (4)	
O2	0.70934 (5)	0.38856 (15)	0.20630 (16)	0.0332 (3)	
O3	0.86165 (6)	−0.36494 (16)	0.1328 (2)	0.0442 (4)	
O4	0.93566 (5)	−0.26941 (15)	0.30322 (18)	0.0383 (4)	
N1	0.72949 (6)	0.25526 (19)	−0.0044 (2)	0.0341 (4)	
H1N	0.7239	0.2407	−0.1056	0.041*	
C1	0.51676 (8)	0.2878 (2)	0.0816 (3)	0.0355 (5)	
C2	0.53176 (8)	0.4194 (2)	0.1302 (3)	0.0375 (5)	
H2	0.5094	0.4775	0.1883	0.045*	
C3	0.57995 (8)	0.4671 (2)	0.0936 (2)	0.0341 (5)	
H3	0.5908	0.5576	0.1286	0.041*	
C4	0.61212 (7)	0.3842 (2)	0.0069 (2)	0.0298 (4)	
C5	0.59606 (8)	0.2516 (2)	−0.0420 (3)	0.0375 (5)	
H5	0.6179	0.1942	−0.1025	0.045*	
C6	0.54833 (9)	0.2019 (2)	−0.0035 (3)	0.0404 (5)	
H6	0.5376	0.1103	−0.0352	0.048*	
C7	0.66491 (7)	0.4385 (2)	−0.0328 (2)	0.0315 (4)	
H7	0.6672	0.5409	−0.0051	0.038*	
C8	0.64981 (9)	0.5251 (2)	−0.2903 (3)	0.0417 (5)	
H8A	0.6130	0.5220	−0.2700	0.050*	
H8B	0.6547	0.5010	−0.4025	0.050*	
C9	0.66839 (9)	0.6681 (3)	−0.2614 (3)	0.0446 (6)	
C10	0.68291 (11)	0.7840 (3)	−0.2365 (3)	0.0575 (7)	
H10	0.6946	0.8773	−0.2164	0.069*	
C11	0.70398 (7)	0.3566 (2)	0.0662 (2)	0.0291 (4)	
C12	0.76667 (8)	0.1662 (3)	0.0785 (3)	0.0380 (5)	
H12A	0.7492	0.0829	0.1222	0.046*	
H12B	0.7817	0.2200	0.1678	0.046*	
C13	0.80847 (8)	0.1170 (3)	−0.0268 (3)	0.0387 (5)	
H13A	0.7936	0.0687	−0.1202	0.046*	
H13B	0.8280	0.1994	−0.0639	0.046*	
C14	0.84327 (8)	0.0174 (2)	0.0610 (2)	0.0346 (5)	
C15	0.83530 (8)	−0.1280 (2)	0.0534 (3)	0.0355 (5)	
H15	0.8081	−0.1637	−0.0096	0.043*	
C16	0.86616 (8)	−0.2208 (2)	0.1356 (3)	0.0343 (5)	
C17	0.90637 (7)	−0.1685 (2)	0.2301 (2)	0.0329 (5)	
C18	0.91396 (8)	−0.0250 (2)	0.2401 (3)	0.0376 (5)	
H18	0.9407	0.0113	0.3046	0.045*	
C19	0.88242 (8)	0.0672 (2)	0.1556 (3)	0.0389 (5)	
H19	0.8880	0.1661	0.1633	0.047*	
C20	0.81997 (9)	−0.4224 (3)	0.0456 (3)	0.0503 (6)	
H20A	0.7882	−0.3864	0.0881	0.075*	
H20B	0.8206	−0.5256	0.0538	0.075*	
H20C	0.8225	−0.3948	−0.0653	0.075*	
C21	0.97493 (9)	−0.2226 (3)	0.4095 (3)	0.0428 (5)	
H21A	0.9884	−0.3049	0.4687	0.051*	
H21B	0.9603	−0.1563	0.4864	0.051*	
C22	1.01689 (8)	−0.1525 (2)	0.3292 (3)	0.0417 (5)	
C23	1.05017 (10)	−0.0922 (3)	0.2672 (4)	0.0542 (7)	
H23	1.0770	−0.0435	0.2173	0.065*	

### Atomic displacement parameters (Å^2^)

**Table:**

	*U*^11^	*U*^22^	*U*^33^	*U*^12^	*U*^13^	*U*^23^
Cl1	0.0349 (3)	0.0530 (4)	0.0753 (5)	−0.0051 (3)	0.0082 (3)	0.0229 (3)
O1	0.0426 (9)	0.0383 (8)	0.0318 (8)	0.0099 (7)	0.0019 (6)	0.0045 (6)
O2	0.0363 (8)	0.0359 (8)	0.0272 (8)	0.0032 (6)	−0.0033 (6)	−0.0002 (6)
O3	0.0406 (9)	0.0346 (8)	0.0572 (10)	−0.0052 (7)	−0.0040 (8)	−0.0044 (7)
O4	0.0323 (8)	0.0362 (8)	0.0462 (9)	0.0008 (6)	−0.0047 (7)	0.0013 (7)
N1	0.0321 (9)	0.0464 (10)	0.0238 (9)	0.0094 (8)	−0.0022 (7)	0.0002 (8)
C1	0.0289 (10)	0.0399 (12)	0.0376 (12)	−0.0010 (9)	−0.0011 (9)	0.0130 (10)
C2	0.0335 (11)	0.0385 (12)	0.0406 (12)	0.0072 (9)	0.0060 (9)	0.0049 (10)
C3	0.0329 (11)	0.0322 (11)	0.0370 (12)	0.0015 (9)	−0.0007 (9)	0.0025 (9)
C4	0.0278 (10)	0.0342 (10)	0.0272 (10)	0.0023 (8)	−0.0040 (8)	0.0065 (8)
C5	0.0370 (12)	0.0410 (12)	0.0347 (12)	0.0057 (9)	0.0025 (9)	−0.0018 (9)
C6	0.0408 (12)	0.0361 (12)	0.0440 (13)	−0.0038 (10)	−0.0043 (10)	0.0021 (10)
C7	0.0285 (10)	0.0390 (11)	0.0270 (10)	0.0053 (8)	−0.0022 (8)	−0.0002 (9)
C8	0.0417 (12)	0.0439 (13)	0.0394 (13)	0.0066 (10)	−0.0042 (10)	0.0133 (10)
C9	0.0429 (13)	0.0469 (14)	0.0439 (14)	0.0045 (11)	−0.0002 (10)	0.0175 (11)
C10	0.0650 (18)	0.0500 (16)	0.0574 (17)	−0.0014 (13)	−0.0046 (14)	0.0125 (13)
C11	0.0238 (9)	0.0344 (11)	0.0290 (11)	−0.0015 (8)	0.0015 (8)	0.0046 (8)
C12	0.0341 (11)	0.0478 (13)	0.0321 (12)	0.0116 (10)	0.0027 (9)	0.0022 (10)
C13	0.0334 (11)	0.0467 (13)	0.0362 (12)	0.0098 (10)	0.0053 (9)	0.0029 (10)
C14	0.0282 (10)	0.0419 (12)	0.0339 (11)	0.0056 (9)	0.0063 (8)	−0.0003 (9)
C15	0.0260 (10)	0.0448 (12)	0.0355 (12)	0.0007 (9)	0.0004 (9)	−0.0061 (10)
C16	0.0300 (10)	0.0342 (11)	0.0389 (12)	−0.0003 (9)	0.0049 (9)	−0.0039 (9)
C17	0.0262 (10)	0.0369 (11)	0.0356 (11)	0.0040 (8)	0.0036 (8)	−0.0006 (9)
C18	0.0273 (10)	0.0384 (11)	0.0471 (13)	−0.0005 (9)	−0.0014 (9)	−0.0071 (10)
C19	0.0295 (11)	0.0331 (11)	0.0541 (14)	0.0025 (9)	0.0034 (10)	−0.0022 (10)
C20	0.0419 (13)	0.0477 (14)	0.0611 (16)	−0.0150 (11)	0.0014 (12)	−0.0080 (12)
C21	0.0380 (12)	0.0478 (13)	0.0426 (13)	0.0008 (10)	−0.0064 (10)	0.0044 (11)
C22	0.0317 (11)	0.0387 (12)	0.0542 (14)	0.0063 (10)	−0.0086 (10)	0.0004 (11)
C23	0.0346 (13)	0.0445 (14)	0.083 (2)	0.0032 (11)	−0.0008 (13)	0.0063 (14)

### Geometric parameters (Å, º)

**Table:**

Cl1—C1	1.742 (2)	C8—H8B	0.9900
O1—C7	1.405 (2)	C9—C10	1.181 (4)
O1—C8	1.432 (2)	C10—H10	0.9500
O2—C11	1.232 (2)	C12—C13	1.506 (3)
O3—C16	1.371 (3)	C12—H12A	0.9900
O3—C20	1.423 (3)	C12—H12B	0.9900
O4—C17	1.371 (2)	C13—C14	1.505 (3)
O4—C21	1.432 (3)	C13—H13A	0.9900
N1—C11	1.323 (3)	C13—H13B	0.9900
N1—C12	1.464 (3)	C14—C19	1.380 (3)
N1—H1N	0.8800	C14—C15	1.394 (3)
C1—C2	1.369 (3)	C15—C16	1.379 (3)
C1—C6	1.378 (3)	C15—H15	0.9500
C2—C3	1.389 (3)	C16—C17	1.409 (3)
C2—H2	0.9500	C17—C18	1.376 (3)
C3—C4	1.379 (3)	C18—C19	1.396 (3)
C3—H3	0.9500	C18—H18	0.9500
C4—C5	1.386 (3)	C19—H19	0.9500
C4—C7	1.528 (3)	C20—H20A	0.9800
C5—C6	1.388 (3)	C20—H20B	0.9800
C5—H5	0.9500	C20—H20C	0.9800
C6—H6	0.9500	C21—C22	1.469 (3)
C7—C11	1.529 (3)	C21—H21A	0.9900
C7—H7	1.0000	C21—H21B	0.9900
C8—C9	1.460 (4)	C22—C23	1.179 (3)
C8—H8A	0.9900	C23—H23	0.9500
			
C7—O1—C8	112.75 (16)	C13—C12—H12A	109.1
C16—O3—C20	117.13 (18)	N1—C12—H12B	109.1
C17—O4—C21	117.70 (17)	C13—C12—H12B	109.1
C11—N1—C12	122.83 (17)	H12A—C12—H12B	107.8
C11—N1—H1N	118.6	C14—C13—C12	110.27 (18)
C12—N1—H1N	118.6	C14—C13—H13A	109.6
C2—C1—C6	121.4 (2)	C12—C13—H13A	109.6
C2—C1—Cl1	119.05 (17)	C14—C13—H13B	109.6
C6—C1—Cl1	119.50 (18)	C12—C13—H13B	109.6
C1—C2—C3	119.3 (2)	H13A—C13—H13B	108.1
C1—C2—H2	120.3	C19—C14—C15	118.4 (2)
C3—C2—H2	120.3	C19—C14—C13	121.1 (2)
C4—C3—C2	120.5 (2)	C15—C14—C13	120.4 (2)
C4—C3—H3	119.7	C16—C15—C14	121.3 (2)
C2—C3—H3	119.7	C16—C15—H15	119.4
C3—C4—C5	119.24 (19)	C14—C15—H15	119.4
C3—C4—C7	119.81 (19)	O3—C16—C15	125.2 (2)
C5—C4—C7	120.94 (19)	O3—C16—C17	115.09 (19)
C4—C5—C6	120.7 (2)	C15—C16—C17	119.7 (2)
C4—C5—H5	119.7	O4—C17—C18	125.51 (19)
C6—C5—H5	119.7	O4—C17—C16	115.16 (18)
C1—C6—C5	118.8 (2)	C18—C17—C16	119.3 (2)
C1—C6—H6	120.6	C17—C18—C19	120.1 (2)
C5—C6—H6	120.6	C17—C18—H18	119.9
O1—C7—C4	111.42 (16)	C19—C18—H18	119.9
O1—C7—C11	109.91 (16)	C14—C19—C18	121.2 (2)
C4—C7—C11	108.39 (16)	C14—C19—H19	119.4
O1—C7—H7	109.0	C18—C19—H19	119.4
C4—C7—H7	109.0	O3—C20—H20A	109.5
C11—C7—H7	109.0	O3—C20—H20B	109.5
O1—C8—C9	112.75 (19)	H20A—C20—H20B	109.5
O1—C8—H8A	109.0	O3—C20—H20C	109.5
C9—C8—H8A	109.0	H20A—C20—H20C	109.5
O1—C8—H8B	109.0	H20B—C20—H20C	109.5
C9—C8—H8B	109.0	O4—C21—C22	112.97 (19)
H8A—C8—H8B	107.8	O4—C21—H21A	109.0
C10—C9—C8	179.0 (3)	C22—C21—H21A	109.0
C9—C10—H10	180.0	O4—C21—H21B	109.0
O2—C11—N1	124.40 (18)	C22—C21—H21B	109.0
O2—C11—C7	118.06 (18)	H21A—C21—H21B	107.8
N1—C11—C7	117.53 (17)	C23—C22—C21	177.8 (3)
N1—C12—C13	112.59 (17)	C22—C23—H23	180.0
N1—C12—H12A	109.1		
			
C6—C1—C2—C3	−0.3 (3)	C11—N1—C12—C13	−149.7 (2)
Cl1—C1—C2—C3	−179.05 (17)	N1—C12—C13—C14	−175.73 (19)
C1—C2—C3—C4	1.2 (3)	C12—C13—C14—C19	−84.1 (3)
C2—C3—C4—C5	−0.7 (3)	C12—C13—C14—C15	93.3 (3)
C2—C3—C4—C7	179.77 (19)	C19—C14—C15—C16	−1.2 (3)
C3—C4—C5—C6	−0.5 (3)	C13—C14—C15—C16	−178.71 (19)
C7—C4—C5—C6	178.97 (19)	C20—O3—C16—C15	−3.9 (3)
C2—C1—C6—C5	−0.9 (3)	C20—O3—C16—C17	176.77 (19)
Cl1—C1—C6—C5	177.82 (17)	C14—C15—C16—O3	−178.8 (2)
C4—C5—C6—C1	1.3 (3)	C14—C15—C16—C17	0.5 (3)
C8—O1—C7—C4	76.5 (2)	C21—O4—C17—C18	5.6 (3)
C8—O1—C7—C11	−163.30 (17)	C21—O4—C17—C16	−175.78 (18)
C3—C4—C7—O1	−130.98 (19)	O3—C16—C17—O4	1.2 (3)
C5—C4—C7—O1	49.5 (2)	C15—C16—C17—O4	−178.20 (18)
C3—C4—C7—C11	108.0 (2)	O3—C16—C17—C18	179.9 (2)
C5—C4—C7—C11	−71.5 (2)	C15—C16—C17—C18	0.5 (3)
C7—O1—C8—C9	65.9 (2)	O4—C17—C18—C19	177.8 (2)
C12—N1—C11—O2	1.5 (3)	C16—C17—C18—C19	−0.8 (3)
C12—N1—C11—C7	−177.93 (19)	C15—C14—C19—C18	1.0 (3)
O1—C7—C11—O2	161.04 (17)	C13—C14—C19—C18	178.4 (2)
C4—C7—C11—O2	−77.0 (2)	C17—C18—C19—C14	0.0 (3)
O1—C7—C11—N1	−19.5 (3)	C17—O4—C21—C22	−69.6 (3)
C4—C7—C11—N1	102.5 (2)		

### Hydrogen-bond geometry (Å, º)

Cg2 is the centroid of the C14–C19 ring.

**Table:**

*D*—H···*A*	*D*—H	H···*A*	*D*···*A*	*D*—H···*A*
N1—H1*N*···O2^i^	0.88	2.04	2.850 (2)	152
C10—H10···O2^ii^	0.95	2.35	3.218 (3)	152
C12—H12*A*···O1^iii^	0.99	2.53	3.232 (3)	128
C20—H20*C*···*Cg*2^iv^	0.98	2.84	3.709 (3)	148

Symmetry codes: (i) *x*, −*y*+1/2, *z*−1/2; (ii) *x*, −*y*+3/2, *z*−1/2; (iii) *x*, −*y*+1/2, *z*+1/2; (iv) *x*, −*y*−1/2, *z*−1/2.
