# Supplementary figures and images for: Crystal structure of mandipropamid
Source: Acta Crystallogr E Crystallogr Commun. 2015 Sep 12;71(Pt 10):o727–8. doi: 10.1107/S2056989015016643 (PMC4647438; doi:10.1107/S2056989015016643)

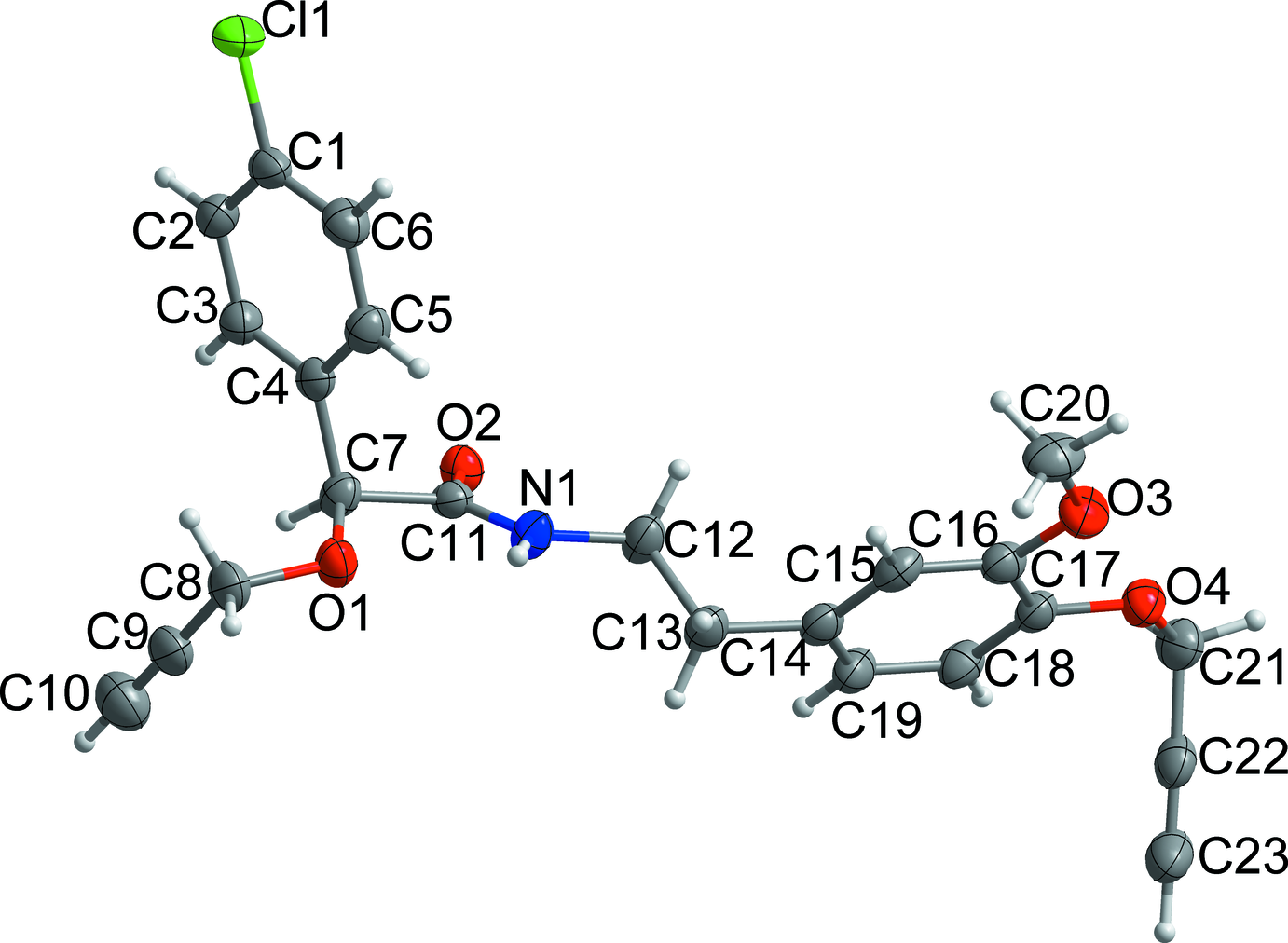

Supplement: Supplementary file 4 [file e-71-0o727-fig1.tif]

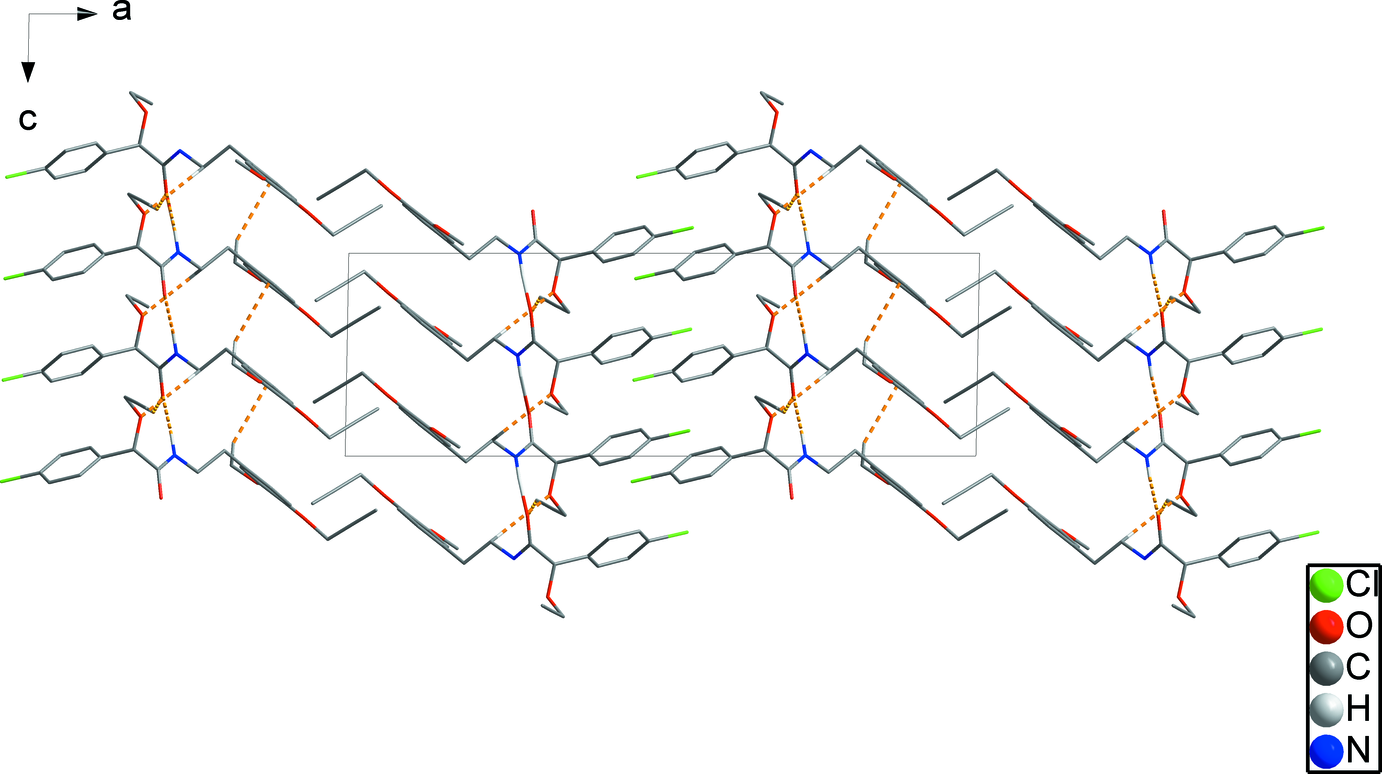

Supplement: Supplementary file 5 [file e-71-0o727-fig2.tif]
